# *Paraboea wenshanensis*, a new species of Gesneriaceae from Yunnan, China

**DOI:** 10.3897/phytokeys.95.21586

**Published:** 2018-02-09

**Authors:** De-Ming He, Yan-Fei Feng, Fu-Zhuan Pan, Xin Hong, Fang Wen

**Affiliations:** 1 Administration of Wenshan National Nature Reserve, Wenshan Zhuang and Miao Autonomous Prefecture, CN-663000, Yunnan, China; 2 School of Resources and Environmental Engineering, Anhui University, CN-230601, Hefei, China; 3 Guangxi Key Laboratory of Plant Conservation and Restoration Ecology in Karst Terrain, Guangxi Institute of Botany, Guangxi Zhuang Autonomous Region and Chinese Academy of Sciences, CN-541006 Guilin, China

**Keywords:** Limestone flora, karst, new taxon

## Abstract

*Paraboea wenshanensis* is a new species of Gesneriaceae from Yunnan, China and is described and illustrated here. It is morphologically similar to *P. angustifolia*, *P. martinii* and *P. glutinosa*, but the congeners of this new taxon can be distinguished by several salient characters. A description of *P. wenshanensis*, together with illustrations and photographs, a distribution map and conservation assessment are presented.

## Introduction

Southern and south-western China has extensive areas of karst topography (Chenet al. 2008) so that seed plants in this region show extreme diversity and are endemic on alkalescent limestone substrates (Xu 1993), a typical example being Gesneriaceae (Fang et al. 1995, Wang et al. 1998, Li and Wang 2004, Wei et al. 2010, Ai et al. 2015).

*Paraboea* (C.B. Clarke) Ridley has recently been redefined to accommodate *Phylloboea* Benth and *Trisepalum* C.B. Clarke (Middleton et al. 2010, Puglisi et al. 2011), distributed from south to southwest China, Indo-China Peninsula to Malaysia, Indonesia and the Philippines (Burtt 1984, Xu and Burtt 1991, Xu et al. 2008, Triboun and Middleton 2012). Many new taxa of this genus have been discovered and published in recent years (Chen et al. 2008, Kiew 2010, Wei et al. 2010, Chen et al. 2012, Wen et al. 2013, Wen and Wei 2016). *Paraboea* is restricted to Asia and includes about 130+ species (Weber 2004, Wen and Wei 2016).

In 2009, one of the authors (WF) encountered a *Paraboea* species with last year’s fruits when collecting plants specimens endemic to karst landforms in Wenshan Zhuang and Miao Autonomous Prefecture, Yunnan Province. Then, in the course of floristic surveys in Wenshan National Nature Reserve between 2012 and 2014, the same species was again collected by the authors. After thorough comparisons of diagnostic morphological and anatomical features of similar taxa from China, Vietnam and Thailand (Barnett 1961, Wang 1990, 1998, Xu and Burtt 1991, Xu 1994, Pham-Hoang 2000, Burtt 2001, Li and Wang 2004, Xu et al. 2008), it was concluded that it was a species new to science and thus it is described and illustrated here.

## Material and methods

Measurements and morphological character assessments of the putative new species were undertaken and described using specimens worked on by the current authors and living material observed in the field and at the Gesneriad Conservation Centre of China. All available specimens of *Paraboea* stored in the following herbaria in China, Vietnam, the United States and the United Kingdom were examined (codes according to Thiers 2015): E, GH, HN, IBK, K, KUN, MO, PE, PH, US and VNMN. In addition, images of other type specimens were obtained from Tropicos (http://www.tropicos.org), JSTOR Global Plants (http://plants.jstor.org) and the International Plant Names Index (http://www.ipni.org). All morphological characters were studied under dissecting microscopes and are described using the terminology presented by Wang et al. (1998).

## Taxonomic treatment

### Paraboea wenshanensis

Taxon classificationPlantaeLamialesGesneriaceae

X.Hong & F.Wen sp. nov.

urn:lsid:ipni.org:names:60476045-2

Figures 1 and 2

#### Diagnosis.

*Paraboea wenshanensis* is similar to *P. martinii* (H. Lév. & Vaniot) B.L. Burtt and *P. glutinosa* (Hand.-Mazz.) K.Y. Pan in having similar corolla shape and colour, but can be distinguished by its oblong-ovate to elliptic leaf blade, crenate margin, lateral veins 4–8 on each side of midrib, petiole subsessile or up to 3 cm long, broadly obovate, glabrous bracts, 6–8 mm long, glabrous membranous calyx and capitate staminodes. It also morphologically resembles *P. angustifolia* Yan Liu & W.B. Xu, but can be easily distinguished by the oblong-ovate to elliptic leaf blade, broadly obovate, glabrous bracts, oblong to oblanceolate, glabrous membranous calyx, sparsely glandular puberulent filaments; capitate staminodes and twisted capsule. A morphological comparison between *P. wenshanensis* and congeners: *P. angustifolia*, *P. martinii* and *P. glutinosa* is provided in Table 1. (see also Fig. 3).

**Figure 1.**
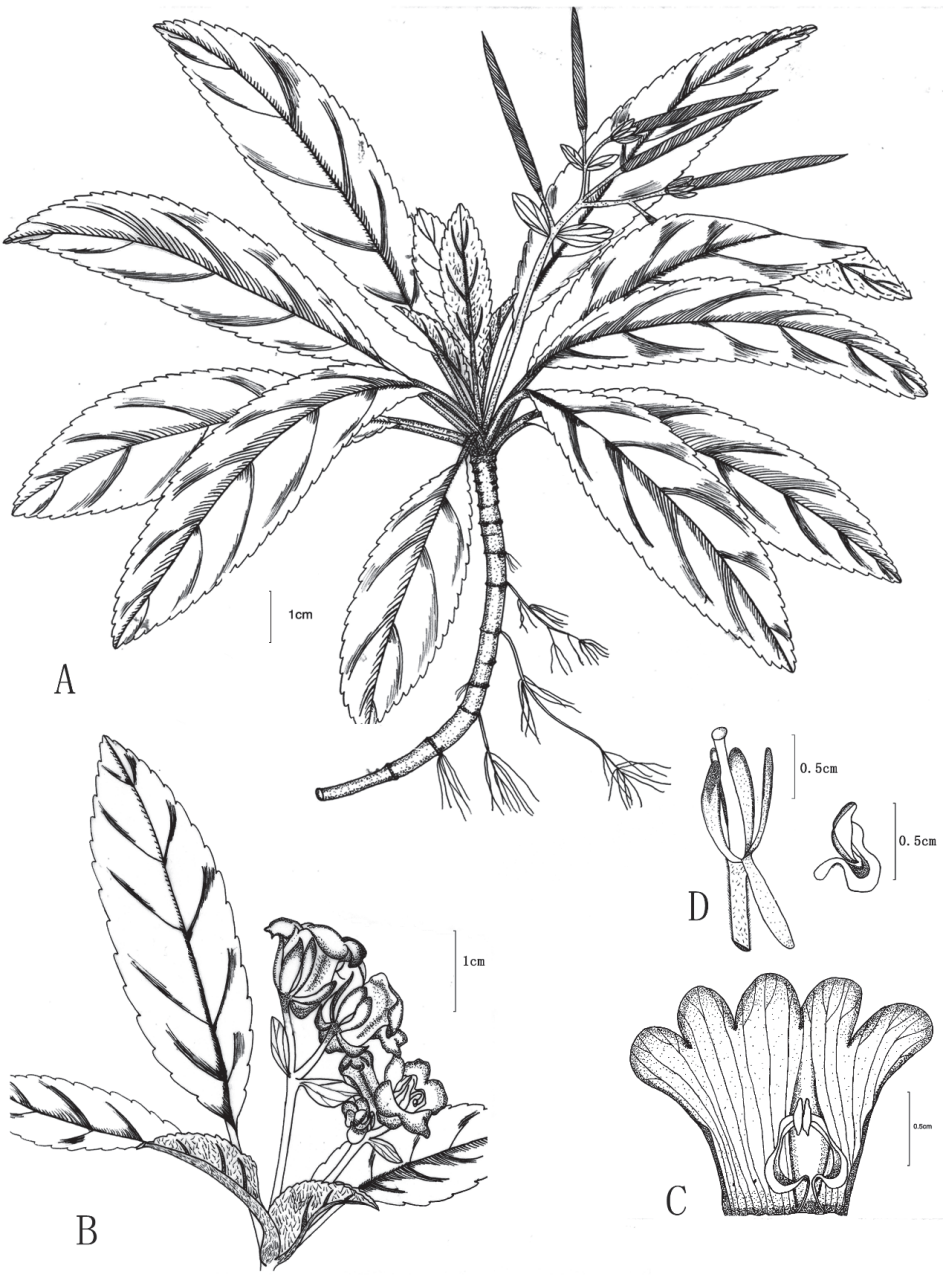
*Paraboea wenshanensis* X.Hong & F.Wen. **A** Habitat in fruiting period **B** Inflorescences **C** Dissection of a flower showing corolla, stamens and staminodes **D** Calyx and pistil, stamen (showing the glandular–puberulous, inflated and strongly geniculate).

**Figure 2.**
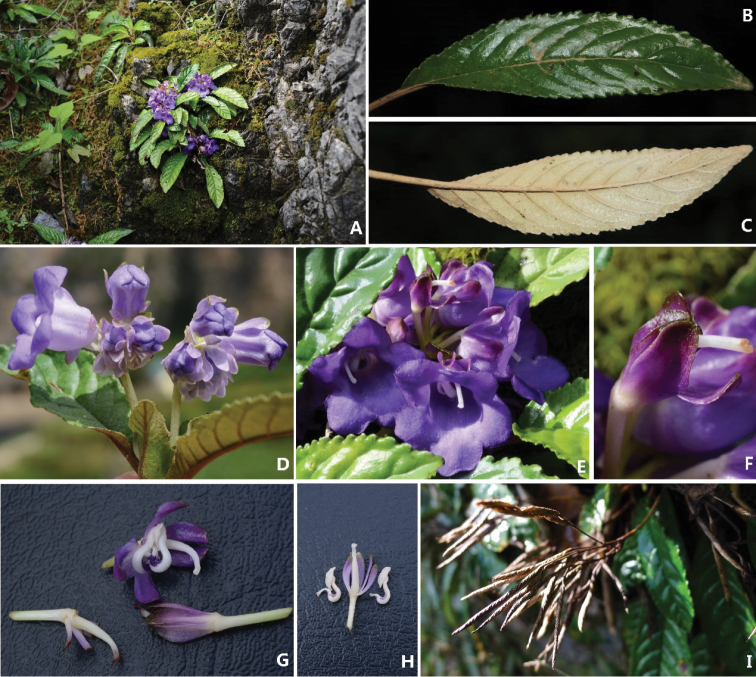
*Paraboea wenshanensis* X.Hong & F.Wen. **A** Habitat **B** Adaxial surface view of leaf blade **C** Abaxial surface view of leaf blade **D** Cyme with flowers, showing wide campanulate **E** Frontal view of corolla **F** Calyx lobes **G** Opened corolla for showing stamens and pistil **H** Pistil with calyx lobes and stamens, showing anthers and strongly geniculate filaments **I** Infructescence with many capsules.

**Table 1.** Diagnostic character differences amongst *Paraboea wenshanensis* sp. nov., *P. angustifolia*, *P. martinii* and *P. glutinosa*.

Characters	*P. wenshanensis*	*P. angustifolia*	*P. martinii*	*P. glutinosa*
**Petiole**	subsessile or up to 3 cm long	subsessile or up to 2 cm long	2–10 cm long	3–7 cm long
**Shape of leaf blade**	oblong-ovate to elliptic	linear-oblanceolate	elliptic to ovate or oblanceolate	obovate to elliptic, ovate or oblong
**Margin of leaf blade**	crenate	serrulate	serrulate to crenulate	serrate to subentire
**Number of lateral veins on each side of midrib**	4–8	4–8	7–11	10–14
**Shape of Bracts**	broadly obovate	linear-lanceolate	lanceolate to ovate,	narrowly ovate to obovate
**Indumentum of bracts**	glabrous	outside pannose	outside pannose	outside pannose
**Shape of Calyx**	oblong to oblanceolate	linear-lanceolate	narrowly oblong to narrowly triangular	lanceolate to narrowly triangular
**Texture of calyx**	membranous	thick papery	papery	papery
**Indumentum of calyx**	glabrous	pannose	sparsely puberulent	glandular puberulent to glabrous
**Indumentum of filaments**	sparsely glandular puberulent	glabrous	bearded	glabrous to glandular puberulent
**Staminodes**	capitate, ca. 0.2 mm long	linear, ca. 4 mm long,	linear, ca. 3 mm long,	linear, 1.2–2 mm long
**Capsule**	twisted	straight	twisted	twisted

**Figure 3.**
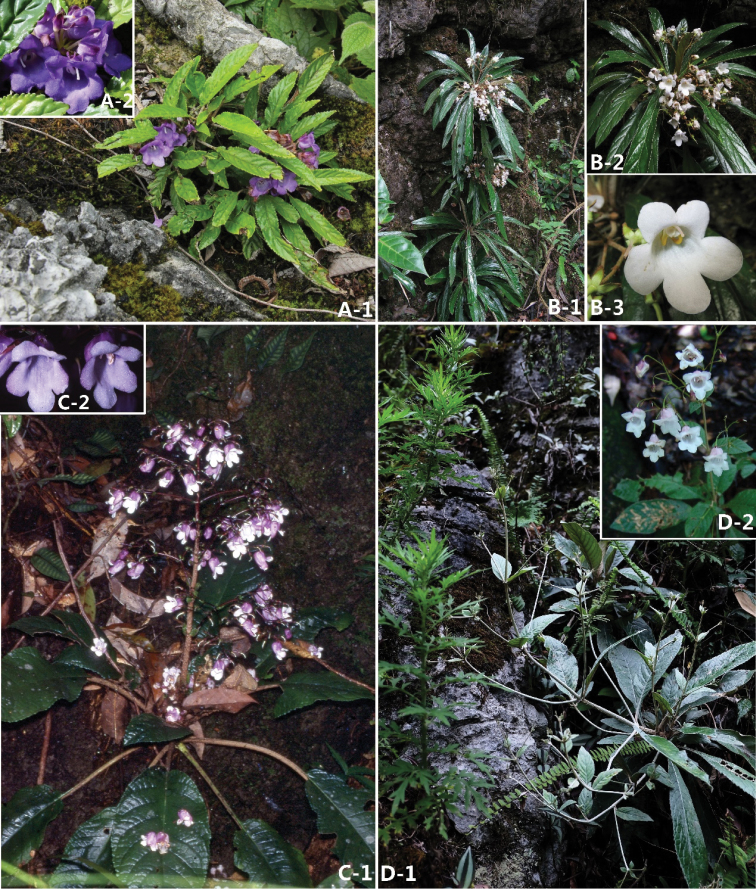
**A**:*Paraboea wenshanensis* X.Hong & F.Wen. **A-1** plant in habitat **A-2** corolla face view **B** *P. angustifolia* Yan Liu & W.B. Xu **B-1** habitat **B-2** flowering habit, **B-3** corolla face view **C** *P. martinii* (Lévl.) Burt **C-1** flowering habit **C-2** corolla face view **D** *P. glutinosa* (Handel-Mazzetti) K. Y. Pan **D-1** plant in habitat **D-2** inflorescense, showing corolla face view.

#### Type.

CHINA. Yunnan Province: Shaka County, Gumu Town, Wenshan Zhuang and Miao Autonomous Prefecture, 23°9'22.5"N, 104°12'16.59"E, a.s.l. 1,500 m, 4 Jul 2014, flowering, *D.M. He & Y.F. Feng WSLJS646* (holotype: KUN; isotype: AHU, IBK).

#### Description.

*Terrestrial* lithophilic, perennial rosulate herbs. *Stems* subterete, 5–10 cm long, 5–9 mm in diameter. *Leaves* 6–20, congested at the apex of the stem, subsessile or up to 3 cm long, leaf blade oblong-ovate to elliptic, (5–)8–18 × 1–3 cm, coriaceous, bases strongly oblique and asymmetrically attenuate, margins crenate sometimes with a woolly strip, apices acute to obtuse, upper leaf surfaces with arachnoid covering when young, becoming glabrescent with age, lower leaf densely appressed greyish arachnoid hairs; lateral veins 4–8 on each side of midrib, convex and densely appressed brown to greyish woolly hairs along the abaxial veins. *Cymes* dichotomous, axillary or subterminal, dichasia 1–3(–5), (2–)4–16-flowered; peduncles 3–10 cm long, ca. 5 mm in diameter, sparsely greyish matted woolly hairs, green; bracts 2, opposite, broadly obovate, 7–9 × ca. 5 mm, margins entire, apices blunted to obtuse, glabrous, whitish to purple; pedicels 0.8–1 cm long, ca. 2 mm in diameter, sparsely greyish matted woolly, greenish. *Calyx* membranous, 5-parted to the base, lobes oblong to oblanceolate, 6–8 × ca. 2.6 mm, glabrous, margins entire, apex obtuse or rounded, white to purplish. *Corolla* zygomorphic, 1–1.5 cm long, purple outside, bluish or purplish inside, glabrous; tube obliquely wide campanulate, 0.6–1.2 cm long, ca. 1cm in diameter at the mouth; the limb two-lipped; adaxial lip 2-lobed to near base, lobes semi-orbicular, apex rounded, 2–4 × ca. 3 mm, abaxial lip 3-lobed to base, central lobe ovate, lateral lobes obliquely ovate, the apex of 3 lower lobes rounded, 5–6 × ca. 3 mm. *Stamens* 2, included, adnate to abaxial side of corolla tube near base; filaments 4–5 mm long, inflated and strongly geniculate on the upper part, wide along its length and narrowly constricted at base, white, sparsely purple glandular–puberulous near the base; anthers dorsifixed, ca. 3.5 mm long; transversely spindle-shaped, coherent at the lateral sides, dehiscing longitudinally, white, glabrous; *staminodes* 2, capitate, ca. 0.2 mm long, adnate to ca. 1.5 cm above the corolla tube base. *Pistil* glabrous; ovary narrowly ovoid to conical, ca. 1 cm long, ca. 1.1 mm in diameter, placentas 4, axile, undivided; style ca. 6 mm long, stigma capitate, with numerous papillae. *Capsule* linear, spirally twisted, ca. 3 cm long, 0.4–0.6 cm in diameter, glabrous, slightly curved, dehiscing loculicidally to base.

#### Etymology.

The specific epithet is derived from the type locality, Wenshan National Nature Reserve, Yunnan Province, China.

#### Vernacular name.

Wén Shān Zhǖ Máo Jǜ Tái (Chinese pronunciation); 文山蛛毛苣苔 (Chinese name).

#### Distribution and habitat.

To date, *Paraboea wenshanensis* is locally abundant and endemic to south-western China, from type locality: Wenshan Nature Reserve, Wenshan Zhuang and Miao Autonomous Prefecture, Yunnan province. This species grows on moist shady cliffs of limestone hills, at an elevation of 1,500 m a.s.l. The average temperature is 14.5 °C, the average annual precipitation has been calculated as ca. 1,022 mm. The forest is a subtropical monsoon climate evergreen broad-leaved forest, with main community types of *Ilex polyneura* (Hand.-Mazz.) S.Y. Hu, *Triadica rotundifolia* (Hemsl.) Esser and *Debregeasia orientalis* C.J. Chen.

#### Conservation status.

Current information for this new species is only known from very few collections and details on the size of the population are known in Wenshan Nature Reserve, where the plants’ protected status is guaranteed. Based on five careful field investigations in the past years, this species appears to be locally abundant. Considering that not enough is known about the population, it is proposed that *Paraboea wenshanensis* should currently be classed as data deficient (DD) (IUCN 2016).

#### Notes.

**Map 1.**
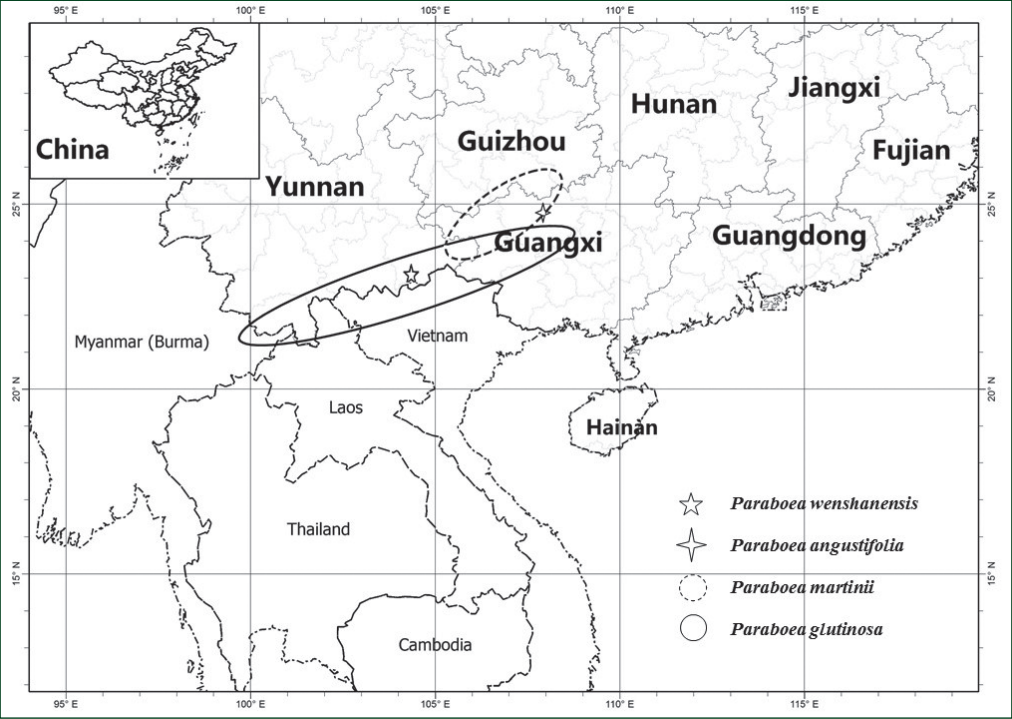
The geographical distribution of *P. wenshanensis* sp. nov., *P. angustifolia*, *P. martinii* and *P. glutinosa*.

The geographical distributions of *P. wenshanensis* and its similar species are identified in Map 1.

## Supplementary Material

XML Treatment for Paraboea wenshanensis

## References

[B1] Ai B Gao Y Zhang X Tao J Kang M Huang H (2015) Comparative transcriptome resources of eleven primulina species, a group of ‘stone plants’ from a biodiversity hot spot. Molecular Ecology Resources 15(3): 619–632. https://doi.org/10.1111/1755-0998.12333 25243665 10.1111/1755-0998.12333

[B2] Barnett EC (1961) Contribution to the Flora of Thailand: LV. Kew Bulletin 15: 249–259. https://doi.org/10.2307/4109363

[B3] Burtt BL (1984) Studies in the Gesneriaceae of the Old World, XLVII: Revised generic concepts for Boea and its allies. Notes from the Royal Botanic Garden Edinburgh 41: 401–452

[B4] Burtt BL (2001) Flora of Thailand: annotated checklist of Gesneriaceae. Thai Forest Bulletin (Botany) 29: 81–109.

[B5] Chen WH Möller M Shui YM Zhang MD (2008) A new species of *Paraboea* (Gesneriaceae) from a karst cave in Guangxi, China and observations on variations in flower and inflorescence architecture. Botanical Journal of the Linnean Society 158: 681–688. https://doi.org/10.1111/j.1095-8339.2008.00873.x

[B6] Chen WH Möller M Zhang MD Shui YM (2012) *Paraboea hekouensis* and *P. manhaoensis*, two new species of Gesneriaceae from China. Annales Botanici Fennici 49(3): 179–187. https://doi.org/10.5735/085.049.0304

[B7] Fang D Qin DH Rao WY Zeng L (1995) New plants of Gesneriaceae from Guangxi and Guizhou of China (Cont. II). Acta Phytotaxonomica Sinica 33: 602–607.

[B8] IUCN (2016) Guidelines for Using the IUCN Red List Categories and Criteria. Version 12. Prepared by the Standards and Petitions Subcommittee. Available from: http://www.iucnredlist.org/documents/RedListGuidelines.pdf [accessed 15 April 2016]

[B9] Kiew R (2010) Two new species of *Paraboea* (Gesneriaceae) from Peninsular Malaysia and Thailand. Edinburgh Journal of Botany 67(2): 209–217. https://doi.org/10.1017/S0960428610000107

[B10] Li ZY Wang YZ (2004) Plants of Gesneriaceae in China. Science and Technology Publishing House: Zhengzhou, Henan, 305–332.

[B11] Middleton DJ Puglisi C Triboun P Möller M (2010) Proposal to conserve *Paraboea* against *Phylloboea* and *Trisepalum* (Gesneriaceae). Taxon 59: 1603.

[B12] Pham–Hoang H (2000) An illustrated flora of Vietnam 3. Youth Publishing House, Ho Chi City, 12–29.

[B13] Puglisi C Middleton DJ Triboun P Möller M (2011) New insights into the relationships between *Paraboea*, *Trisepalum* and *Phylloboea* (Gesneriaceae) and their taxonomic consequences. Taxon 60: 1693–1702.

[B14] Thiers B (2015) [continuously updated] Index herbariorum: a global directory of public herbaria and associated staff. New York Botanical Garden. http://sweetgum.nybg.org/ih/ [accessed 3 Feb 2015]

[B15] Triboun P Middleton DJ (2012) Twenty new species of *Paraboea* (Gesneriaceae) from Thailand. Gardens’ Bulletin Singapore 64(2): 333–370.

[B16] Triboun P (2013) *Paraboea middletonii* (Gesneriaceae), a new species from Thailand. Thai Forest Bulletin (Botany) 41: 45–47.

[B17] Wang WT (1990) Gesneriaceae. In: Wang WT (Ed.) Flora Reipublicae Popularis Sinicae 69. Science Press, Beijing, 460–472.

[B18] Wang WT Pan KY Li ZY Weitzman AL Skog LE (1998) Gesneriaceae. In: Wu ZH Raven PH (Eds) Flora of China, Vol. 18. Science Press, Beijing and Missouri Botanical Garden Press, St. Louis, 362–367.

[B19] Weber A (2004) Gesneriaceae. In: Kubitzki K Kadereit JW (Eds) The Families and Genera of Vascular Plants volume 7: Dicotyledons, Lamiales. Springer, Berlin/Heidelberg, 63–158. https://doi.org/10.1007/978-3-642-18617-2_8

[B20] Wei YG Wen F Möller M Monro A Zhang Q Gao Q Mou HF Zhong SH Cui C (2010) Gesneriaceae of South China. Guangxi Science and Technology Publishing House, Nanning, Guangxi, 608–646.

[B21] Wen F Wei YG (2016) *Paraboea yunfuensis*: a new calcicolous species of Gesneriaceae from Yunfu, Guangdong Province, China. Telopea 19: 125–129.

[B22] Wen F Hong X Chen LY Zhou SB Wei YG (2013) A new species of *Paraboea* (Gesneriaceae) from a karst limestone hill in Southwestern Guangdong, China. Phytotaxa 131(1): 1–8. https://doi.org/10.11646/phytotaxa.131.1.1

[B23] Xu WB Huang YS Wei GF Tan WN Liu Y (2012) *Paraboea angustifolia* (Gesneriaceae): a new species from limestone areas in northern Guangxi, China. Phytotaxa 62(1): 39–43. https://doi.org/10.11646/phytotaxa.62.1.8

[B24] Xu ZR (1993) A study of the limestone forest flora of southern and south-western China: floristics, ecology, conservation and taxonomy. Guihaia Additamentum 4: 5–54.

[B25] Xu ZR (1994) A new species of *Paraboea* Ridley from Thailand. Acta Phytotaxonomica Sinica 32: 359–361.

[B26] Xu ZR Burtt BL (1991) Towards a revision of *Paraboea* (Gesneraceae): I. Edinburgh Journal of Botany 48(1): 1–18. https://doi.org/10.1017/S0960428600003541

[B27] Xu ZR Burtt BL Skog LE Middleton DJ (2008) A revision of *Paraboea* (Gesneriaceae). Edinburgh Journal of Botany 65(2): 161–347. https://doi.org/10.1017/S0960428608005106

